# The Tick-Borne Diseases STING study: Real-time PCR analysis of three emerging tick-borne pathogens in ticks that have bitten humans in different regions of Sweden and the Aland islands, Finland

**DOI:** 10.1080/20008686.2019.1683935

**Published:** 2019-11-02

**Authors:** Samuel Cronhjort, Peter Wilhelmsson, Linda Karlsson, Johanna Thelaus, Andreas Sjödin, Pia Forsberg, Per-Eric Lindgren

**Affiliations:** a Division of Medical Microbiology, Department of Clinical and Experimental Medicine, Linköping University, Linköping, Sweden; b Department of Clinical Microbiology, Jönköping, Region Jönköping County, and the Department of Clinical and Experimental Medicine, Linköping University, Linköping, Sweden; c Division of CBRN Defence and Security, Swedish Defence Research Agency, Umeå, Sweden; d Divison of Infectious Diseases, Department of Clinical and Experimental Medicine, Linköping University, Linköping, Sweden

**Keywords:** *Ixodes ricinus*, *Bartonella* spp., *Francisella tularensis*, *Toxoplasma gondii*, real-time PCR

## Abstract

A milder climate has during the last decade contributed to an increased density and spreading of ixodid ticks, thus enhancing their role as emerging vectors for pathogenic microorganisms in northern Europe. It remains unclear if they contribute to the occurrence of infections caused by the bacteria *Bartonella* spp., *Francisella tularensis* subspecies *holarctica* and the parasite *Toxoplasma gondii* in Sweden and on the Åland islands, Finland. In this study, we want to improve understanding of the tick-borne transmission of these pathogens. Volunteers were recruited at primary healthcare centers. Ticks and blood samples were acquired from participants recruited in 2008 and 2009. Health questionnaires were completed, and medical records were acquired where applicable. Feeding time was estimated and screening of pathogens in the ticks was performed through real-time PCR. Ticks (n = 1849) were of mixed developmental stages: 76 larvae, 1295 nymphs, 426 adults and 52 undetermined. All analyzed ticks were considered negative for these pathogens since the CT-values were all below the detection limit for *Bartonella* spp. (1663 ticks), *Francisella* spp. (1849 ticks) and *Toxoplasma gondii* (1813 ticks). We assume that infections with these pathogens are caused by other transmission pathways within these regions of Sweden and the Åland islands, Finland.

## Introduction

The ixodid ticks are emerging vectors of potential pathogenic microorganisms in northern Europe. Following a milder climate, density and spreading of the ixodid ticks have increased. The prospective Tick-Borne Diseases STING-study (TBD STING) aimed to investigate the risk and factors affecting the onset of tick-borne diseases following a tick bite [1]. Within the TBD STING material the following pathogens have been detected: *Borrelia* spp. [1], Tick-borne encephalitis virus [2], *Anaplasma phagocytophilum* [3], *Rickettsia helvetica* [4], *Candidatus* Neoehrlichia mikurensis [5]. Other assumed tick-borne pathogens that are yet to be detected within the TBD STING study include the bacteria *Bartonella* spp., *Francisella tularensis* and the parasite *Toxoplasma gondii*. These pathogens have been detected within *Ixodes ricinus* in neighboring countries (Table 1) such as Germany (*F. tularensis*) [6], Denmark (*Bartonella* spp.) [7] and Poland (*T. gondii*) [8, 9]. Since infection with *F. tularensis* subspecies (ssp.) *holarctica* is notifiable there is an incidence reported by the Swedish Agency of Public Health [10]; it has varied from 0.9 and 8.7 per 100 000 person-years [11, 12]. Neither *Bartonella* spp. nor *T. gondii* is notifiable and thus the best data available in Sweden are blood donor serology: 16.1% for *Bartonella* spp. [[13]] and 23% for *T. gondii* [14].

**Table 1.** Epidemiology of *Bartonella* spp., *F.* *tularensis* ssp. *holarctica* and *T.* *gondii*.

Pathogen	Occurrence in Sweden	Prevalence in *Ixodes ricinus*	Other vectors and reservoirs
*Bartonella* spp.	16% blood donor seroprevalence [13]	4/661 Denmark [7] Ticks were collected from Danish domestic dogs. Detection through PCR and sequencing.	Cats [15]
*F. tularensis* ssp. *holarctica*	Reported incidence [10] 2015: 8.7/100 000 2017: 0.9/100 000	4-16/1556 Germany [6] Field collected ticks. Detection through MALDI-TOF mass spectrometry or 16S rRNA gene sequencing.	Mosquitos, Rodents, Environment [16, 15]
*T. gondii*	23% blood donor seroprevalence [14]	74/114 Poland [9] Pet and field collected ticks. 33/259 Poland [8] Field collected ticks. Detection through nested PCR.	Warm blooded vertebrates [17]

These emerging pathogens may present with an initial partly similar clinical picture. It is thus of clinical importance to be aware of these unusual tick-borne diseases as antibiotic treatment differs, and untreated or maltreated disease may have severe consequences. Prolonged bacteremia of virulent strains of *Bartonella* may result in endocarditis [15], arthropod transmission of *F. tularensis holarctica* may give rise to typhoidal tularemia (which is a severe systemic disease) [16] and toxoplasmosis in pregnant women may lead to abortion [17]. There are no published data on the prevalence of these pathogens in neither questing nor blood-feeding ticks collected in Sweden except for a minor study [18] that found 167 questing ticks from the Stockholm/Uppsala area in Sweden negative for *Bartonella* spp..

This study aims to investigate the risk of infection by *Bartonella* spp., *F. tularensis* or *T. gondii* after exposure to a tick-bite in different regions of Sweden and the Åland Islands, Finland by determining the prevalence of these pathogens in blood-feeding ticks collected from humans.

## Materials and methods

### Study design of the TBD STING study and collection of samples and preparation

**Figure 1.**
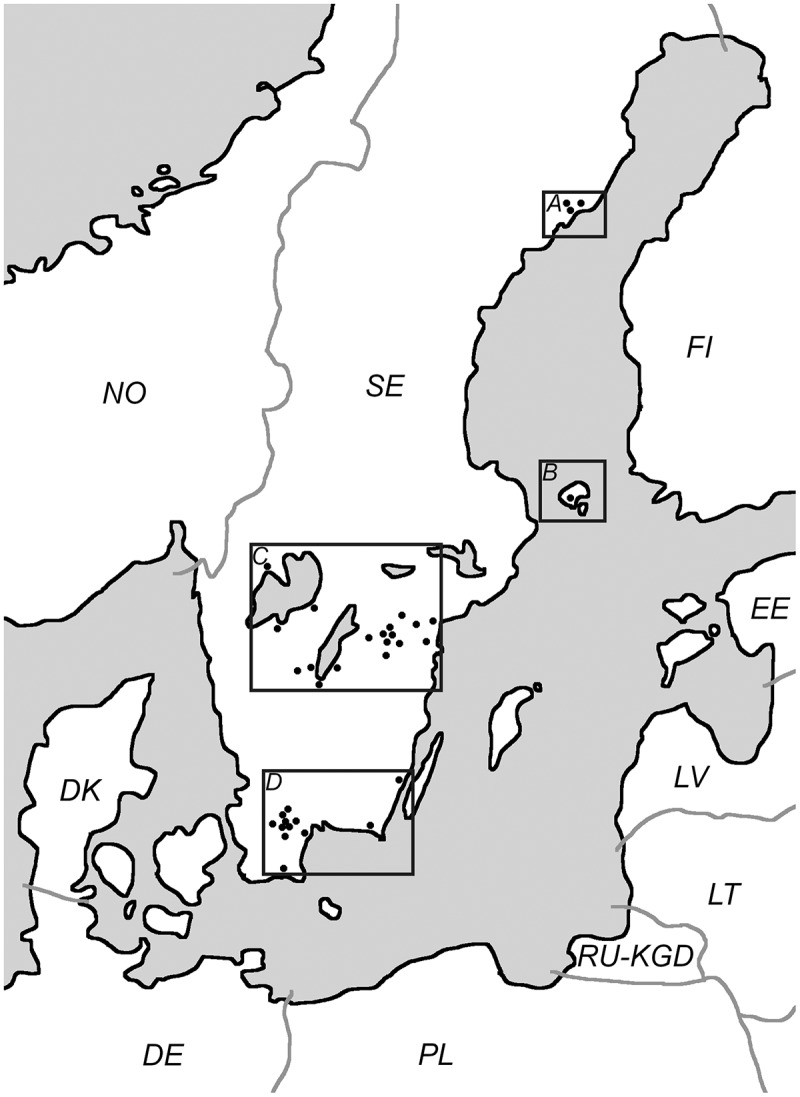
Distribution of the participating primary healthcare centers (n = 34), each black dot represents a primary healthcare center. Regions shown are *A* Northern Sweden (15 ticks), *B* Åland islands, Finland (633 ticks), *C* South Central Sweden (800 ticks) and *D* Southernmost Sweden (401 ticks). Countries coded in accordance with ISO 3166.

Healthy, adult tick-bitten volunteers were recruited at primary healthcare centers through advertisements (Figure 1). Written consent, ticks, blood samples and health questionnaires were collected at inclusion at the first visit to the primary health-care center within 3 days after removal of the tick by the participant or by personnel at the primary health-care center. At the 3 months follow-up visit to the primary healthcare center, blood samples were collected, and a new questionnaire was completed. Medical records were available if the participant had visited the primary healthcare center during the 3-months study period. Samples were collected between 2007 and 2015 and were transported to Linköping University Hospital. Collected ticks were stored at −70°C until analysis and were morphologically determined to be *I. ricinus* and feeding time was estimated [19]. Nucleic acids were extracted, and the complementary (c) DNA was used in real-time PCR analysis of tick-borne pathogens as previously described by Wilhelmsson et al. [1]. In the event of positive real-time PCR screening, confirmation by conventional PCR analysis and sequencing. Successful sequencing would incur examination of the participant’s health questionnaires, serology of the blood samples and analysis of medical records [1].

### Ethics

The TBD STING study was approved by the Regional Ethical review board at Linköping University (M132-06) and by the local Ethics Committee of the Åland Health Care, 2008-05-23.

### Real-time PCR assay for analyses of tick-borne pathogens

#### Bartonella spp.

The individually synthesized cDNA was analyzed in pools of four with 0.5 μl of cDNA from each tick. The forward and reverse primers (Invitrogen Corporation) (Table 2) were used in an SYBR Green assay. The primers target the *ssrA* gene, which is a prokaryotic gene involved in translatory regulation [20]. The positive control used was *Bartonella grahamii* DNA [30 ng/µl], kindly provided by Dr. Martin Andersson, Department of Biology, Lund University, Sweden. Detection limit was determined at [3 pg/µl].

**Table 2.** Real-time PCR assays used to detect *Bartonella* spp., *Francisella* spp. and *T. gondii* in *I. ricinus.*

	Sequence (5ʹ-3ʹ)	Target gene	Size of amplicon (bp)	Reference
*Bartonella* spp.		*ssrA**^1^*	301	Diaz et al. [20]
Forward	GCT ATG GTA ATA AAT GGA CAA TGA AAT AA			
Reverse	GCT TCT GTT GCC AGG TG			
*Francisella* spp.		*sucC**^2^*	125	This study
Forward	AAC TGG CTG ACC TTC AGC AT			
Reverse	GTG GTC GTG GTA AAG CTG GT			
Probe	**FAM**^3^-CCG ATT AGG CTT TCT GCT ACT TCA CGA-**BHQ1**^4^			
Forward (PHV^5^)	GGG CGA ATC ACA GAT TGA ATC			
Reverse (PHV)	GCG GTT CCA AAC GTA CCA A			
Probe (PHV)	**TR**^6^-TTT TTA TGT GTC CGC CAC CAT CTG GAT C-**BHQ1**			
*T. gondii*		*B1**^7^*	98	Lin et al. [22]
Forward	TCC CCT CTG GCG AAA AGT			
Reverse	AGC GTT CGT GGT CAA CTA TCG ATT G			
Probe	**FAM**-TCT GTG CAA CTT TGG TGT ATT CGC AG-**TAMRA**^8^			

One reaction contained 16 µl master mix and 4 µl sample cDNA. The master mix contained 7.2 µl of RNase/DNase free water, 10 µl of Maxima SYBR Green qPCR Master Mix (Thermo Fisher Scientific), 0.4 µl of *Bartonella* forward primer [10 µM] and 0.4 µl of *Bartonella* reverse primer [10 µM].

The PCR assay consisted of one initializing denaturation step at 95°C for 2 min and then 45 cycles of 95°C denaturation for 15 s, 60°C for 60 s and 72°C for 15 s ending with 72°C for 5 min. Immediately after real-time PCR analysis, melting curve analyses were performed by heating to 95°C for 15 s, followed by cooling to 60°C for 1 min, and subsequent heating to 95°C at 0.8°C min-1 with continuous fluorescence recording. A C1000 thermal cycler (Bio-Rad Laboratories Inc, Hercules, CA) was used for the real-time PCR.

#### Francisella spp.

The individually synthesized cDNA was analyzed in pools of four with 1 μl of cDNA from each tick. The primers and the probe used were developed and provided by the Swedish Defense Research Agency (FOI) and target a 125 bp long fragment of the gene coding for the Succinyl-CoA-synthetase beta chain of the *Francisella* genus (Table 2). An internal control consisting of Phocine Herpes Virions [21], kindly provided by the Public Health Agency of Sweden was used to make sure that the PCR reaction was working despite the pool being negative for *Francisella*. The positive control used was *Francisella tularensis* subsp. *holarctica* live vaccine strain (LVS) extracted DNA from culture by FOI [38.3 ng/μl]. Detection limit was determined at [38.3 fg/µl].

One reaction contained 21 µl of master mix and 4 µl sample cDNA. The master mix contained 4.5 µl of RNase/DNase free water, 12.5 µl of Maxima Probe qPCR Master Mix [2X] (Thermo Fisher Scientific), 0.625 µl of *Francisella* spp. forward primer [20 µM], 0.625 µl of *Francisella* spp. reverse primer [20 µM], 0.5 µl of *Francisella* spp. probe [5 µM], 0.625 µl of internal control forward primer [20 µM], 0.625 µl of internal control reverse primer [20 µM], 0.5 µl of Internal control probe [5 µM] and 0.5 µl DNA for the internal control.

The PCR assay consisted of one initializing denaturation at 95°C for 2 min and then 45 cycles of 95°C denaturation for 30 s and 60°C Annealing/Elongation for 60 s. A C1000 thermal cycler (Bio-Rad) was used for the real-time PCR.

#### Toxoplasma gondii

The individually synthesized cDNA was analyzed in pools of four with 0.5 μl of cDNA from each tick. The primers and probe (Invitrogen Corporation) target the *B1* gene of *T. gondii* [22], its function is unknown, but it is present in all investigated genotypes of *T. gondii*. The positive control used was *T. gondii* DNA [445 ng/µl]. It was kindly provided by professor Krzysztof Solarz through Dr Olga Pawelczyk, both the Department of Parasitology, Medical University of Silesia, Poland. Detection limit was determined at [445 pg/µl].

One reaction contained 18 µl of master mix and 2 µl sample cDNA. The master mix contained 6.3 of µl RNase/DNase free water, 10.5 µl of Maxima Universal Master Mix (Thermo Fisher Scientific), 0.4 µl of *T. gondii* forward primer [5 µM], 0.4 µl of *T. gondii* reverse primer [5 µM], 0.4 µl of TaqMan probe [10 µM]. A period of 30 s at 50°C was added to every cycle before the 15 s of denaturation. A C1000 thermal cycler (Bio-Rad) was used for the real-time PCR.

The PCR assay consisted of one initializing denaturation at 95°C for 10 min and then 40 cycles of 95°C denaturation for 15 s and 60°C Annealing/Elongation for 60 s. A C1000 thermal cycler (Bio-Rad) was used for the real-time PCR.

#### Sequencing of positive samples

Any positive pools were repeated individually in the real-time PCR assay and then conventional PCR and sequencing.

Any real-time PCR products matching the positive control in size were sequenced by Macrogen (Amsterdam, Netherlands).

## Results

In total, cDNA from 1 849 ticks (76 larvae, 1295 nymphs, 426 adults, and 52 were unable to be morphologically staged) collected in 2008 and 2009, were available for analysis. The distribution of primary healthcare centers can be seen in Figure 1. All these ticks were analyzed for *Francisella* spp., 1 813 ticks for *T. gondii* and 1 663 ticks for *Bartonella* spp. The variance in number of analyzed ticks is due to limited amounts of cDNA. All samples were negative i.e. all CT-values obtained were below the detection limit.

## Discussion

In Sweden, vector-borne tularemia is mainly associated with mosquitos [23, 24, 25] and it is unknown whether ticks contribute to the presence of the disease in Sweden. However, there are cases of explicitly suspected tick-borne tularemia in Sweden [26, 27]. Despite the high number of ticks analyzed in this study, *Francisella* spp. remains to be detected in ticks collected in Sweden and the Åland islands, Finland. *F. tularensis* has been found in *I. ricinus* collected in Germany within a smaller sample size by Tomaso et al. [6], it is thus likely that the prevalence is lower in the southern regions of Sweden and on the Åland islands, Finland. It is still uncertain how *F. tularensis* is transmitted between ticks; transovarian transmission was shown in early experiments [28, 29] but has not been reproduced with modern methods [30]. The mode of vector-mediated transmission matters since it could affect the geographic distribution of the bacteria; transovarian transmission would likely result in highly concentrated spots of related ticks colonized with *F. tularensis*, while a vector-reservoir transmission could dilute the infested ticks over a greater area. In this study, the participating primary healthcare centers (Figure 1) do not co-localize with the geographic areas known to be endemic for tularemia and there are only 15 ticks collected from the Northern region of Sweden. Since tularemia in Sweden is known to be a focal disease in the middle and northern parts of Sweden [11, 12] it would be a topic for further studies to include additional primary healthcare centers from the areas endemic for the disease.

*Bartonella* spp. and *T. gondii* also remain to be detected in ticks collected in Sweden even though there is seroprevalence among healthy Swedish blood donors (*Bartonella* spp.: 16.1% *T. gondii*: 23%) [14, 13]. Interestingly, these pathogens have been found in *I. ricinus* collected in Poland [9] and Denmark [7]. Due to the larger sample size in this study, it is likely that the prevalence of these pathogens in *I. ricinus* from South Central and Southernmost Sweden and the Åland islands, Finland is considerably lower. Regarding these pathogens, it is possible that the seroprevalence is due to proximity to household cats (cat scratch disease or *T. gondii* oocysts in cat feces) instead of ticks.

A possible explanation for that we were unable to detect *Bartonella* and *T. gondii* in *I. ricinus* collected in Sweden may be the climate change affecting the distribution and prevalence of tick-borne pathogens. The lack of *Bartonella* in Norway [31] and Finland [32] supports that tick-borne *Bartonella* spp. might not tolerate the harsher climate in most of the Nordic countries compared to Denmark [7]. There are no previously published data on the prevalence of *T. gondii* in neither blood-feeding nor questing ticks from the Nordic countries. Up to 4 days may have elapsed between the removal of the tick and freezing at −70°C. It is unlikely that lysis of the bacterial cells within the tick may have affected the quality of the extracted nucleic acids. We have previously detected several pathogens using the above-mentioned approach [1, 3, 4].

In conclusion, the results obtained from this large material indicate that due to the absence of detection, within South Central and Southernmost Sweden and the Åland islands, there is very low risk of tick-borne infection caused by *Bartonella* spp., *F. tularensis* or *T. gondii*. This knowledge may be useful when deciding on antibiotic treatment in conjunction with a tick bite. It is likely that ticks do not significantly contribute to the presence of these pathogens in South Central and Southernmost Sweden and the Åland islands, Finland. It is also possible that the lack of detection is explained by very focal occurrence of the pathogens.

## Data Availability

The data supporting the conclusions of this article are included within the article. Raw data can be shared with researchers upon a specific request.

## Notes

1. Involved in translator regulation.

2. Succinyl-CoA reductase.

3. 6-carboxyfluorescein.

4. Black Hole Quencher.

5. Phocine Herpes Virion.

6. Texas Red fluorescein.

7. Gene of unknown significance.

8. 5-Carboxytetramethylrhodamine.
