# Correction

**DOI:** 10.1080/20008686.2020.1729525

**Published:** 2020-02-21

**Article title**: The Tick-Borne Diseases STING study: Real-time PCR analysis of three emerging tick-borne pathogens in ticks that have bitten humans in different regions of Sweden and the Aland islands, Finland

**Authors**: Cronhjort S, Wilhelmsson P, Karlsson L, Thelaus L, Sjödin A, Forsberg P and Lindgren P-E.

**Journal**: *Infection Ecology & Epidemiology*

**DOI**: 10.1080/20008686.2019.1683935

When the above article was first published online, one of the co-author was missed from the author list.

The corrected authorship and affiliations are:

Samuel Cronhjort^a^, Peter Wilhelmsson^a,b^, Linda Karlsson^c^, Johanna Thelaus^c^, Andreas Sjödin^c^, Olga Pawełczyk^d^, Pia Forsberg^e^ and Per-Eric Lindgren^a,b^

^a^Division of Medical Microbiology, Department of Clinical and Experimental Medicine, Linköping University, Linköping, Sweden

^b^Department of Clinical Microbiology, Jönköping, Region Jönköping County, and the Department of Clinical and Experimental Medicine, Linköping University, Linköping, Sweden

^c^Division of CBRN Defence and Security, Swedish Defence Research Agency, Umeå, Sweden

^d^Department of Parasitology, Faculty of Pharmaceutical Sciences in Sosnowiec, Medical University of Silesia, Katowice, Poland

^e^Divison of Infectious Diseases, Department of Clinical and Experimental Medicine, Linköping University, Linköping, Sweden

